# Effects of Community Assets on Major Health Conditions in England: A Data Analytic Approach

**DOI:** 10.3390/healthcare12161608

**Published:** 2024-08-12

**Authors:** Aristides Moustakas, Linda J. M. Thomson, Rabya Mughal, Helen J. Chatterjee

**Affiliations:** 1 Arts and Sciences, University College London, Gower Street, London WC1E 6BT, UK; linda.thomson@ucl.ac.uk (L.J.M.T.); rabya.mughal@ucl.ac.uk (R.M.); 2 Natural History Museum of Crete, University of Crete, 700 13 Haraklion, Crete, Greece; 3 Division of Biosciences, Department of Genetics, Evolution and Environment, University College London, Gower Street, London WC1E 6BT, UK

**Keywords:** data analytics, multimorbidity, environmental health, healthcare, green space, community assets

## Abstract

Introduction: The broader determinants of health including a wide range of community assets are extremely important in relation to public health outcomes. Multiple health conditions, multimorbidity, is a growing problem in many populations worldwide. Methods: This paper quantified the effect of community assets on major health conditions for the population of England over six years, at a fine spatial scale using a data analytic approach. Community assets, which included indices of the health system, green space, pollution, poverty, urban environment, safety, and sport and leisure facilities, were quantified in relation to major health conditions. The health conditions examined included high blood pressure, obesity, dementia, diabetes, mental health, cardiovascular conditions, musculoskeletal conditions, respiratory conditions, kidney and liver disease, and cancer. Cluster analysis and dendrograms were calculated for the community assets and major health conditions. For each health condition, a statistical model with all community assets was fitted, and model selection was performed. The number of significant community assets for each health condition was recorded. The unique variance, explained by each significant community asset per health condition, was quantified using hierarchical variance partitioning within an analysis of variance model. Results: The resulting data indicate major health conditions are often clustered, as are community assets. The results suggest that diversity and richness of community assets are key to major health condition outcomes. Primary care service waiting times and distance to public parks were significant predictors of all health conditions examined. Primary care waiting times explained the vast majority of the variances across health conditions, with the exception of obesity, which was better explained by absolute poverty. Conclusions: The implications of the combined findings of the health condition clusters and explanatory power of community assets are discussed. The vast majority of determinants of health could be accounted for by healthcare system performance and distance to public green space, with important covariate socioeconomic factors. Emphases on community approaches, significant relationships, and asset strengths and deficits are needed alongside targeted interventions. Whilst the performance of the public health system remains of key importance, community assets and local infrastructure remain paramount to the broader determinants of health.

## 1. Introduction

Addressing the population and health system challenges presented by multiple long-term conditions (i.e., multimorbidity) is a national and global health priority [1,2]. Developing a multimorbidity strategy is identified as a target for several governments including the UK, Denmark, Finland, Germany, and the Netherlands [3,4,5,6]. Around 25% of patients admitted to hospitals in England have more than one health condition, with onset arising several years before admission [3,7]. The age at which people acquire multiple conditions in England is falling, and those living in the most disadvantaged communities can expect to have two or more conditions 10 years earlier than those in the least deprived [8]. The number of people with multiple conditions is high and growing in older adults; it is projected to significantly deteriorate in the near future [2,3]. The way biological, psychological, behavioural, socioeconomic, and environmental factors interact to trigger health conditions is complex, and sufficient knowledge on the best approaches to prevent multiple conditions is generally lacking [2,3]. The importance of deprivation and other common public health risk factors implies that effective prevention merits population-based strategies that combine the environmental, social, and economic determinants of health [9,10]. Reducing health inequalities has been a priority in the UK and elsewhere; however, despite these efforts, progress has been limited [8].

Some conditions are more prevalent than others in people with multiple conditions [2,7] and tend to form clusters. A cluster can be an excess of cases in either space (i.e., geographic cluster) or time (i.e., temporal cluster), or both [11]. The fact that patients often have multiple health conditions implies that understanding their clustering could offer novel insights into patterns, processes, and co-occurrences, as well as designing interventions and setting public health priorities [8]. Whilst studies have found high numbers of potential cluster combinations, there is also a significant proportion of people in no distinct cluster category [12]. The lack of distinct clusters means that an interdisciplinary approach may be more appropriate than treatment based on a single health condition or a specific disease cluster [13,14]. From a statistical perspective, clusters imply correlations between health conditions or community assets, and their unique effect is hard to quantify [15]. As the issue is complex, an ecosystem approach, embracing the complexity and investigating how asset diversity brings about stability, may be applicable [16]. Data analytical approaches may provide novel insight into understanding patterns, relationships, and a bigger picture, despite not providing mechanisms or causality [17,18].

Community assets, such as healthcare facilities, environmental conditions, green space, cultural organisations, living conditions, financial conditions, infrastructure, and safety, are key factors influencing health conditions [19]. These assets are often unequally distributed across locations, potentially causing deprivation. More often than not, people in disadvantaged areas have poorer access to health facilities and green space; their environments tend to be more polluted, overpopulated, and less safe [20,21]. However, the relative importance of community assets to each major health condition, as well as their combined effect, is not well known. This lack of knowledge derives from methodological limitations; results can be difficult to compare and the findings contradictory, and there is a scarcity of data, as well as difficulties in making comparisons between different data sources and regions [22]. In addition, community assets are diverse, spanning green space and pollution to healthcare system metrics; consequently, their effects across health conditions are hard to quantify.

The environment is of fundamental importance to health conditions [23]. Exposure to air pollution is a serious health risk factor linked to among other illnesses, respiratory problems, heart disease, and lung cancer [24]. There is evidence that the natural environment and green space, such as public parks or private gardens, improve cognition and cardiovascular conditions [25,26]. Moreover, the natural environment and green space reduce stress and anxiety, and they have a positive effect on mental health [27]. The way the urban environment is formed and partitioned among people also has a pronounced effect on health conditions [28]. Household overcrowding can lead to poor childcare, mental health problems, exposure to respiratory hazards and infectious diseases and, ultimately, reduce life expectancy [29]. Homelessness or rough sleeping is closely connected to declines in physical and mental health. Homeless people suffer high rates of infectious diseases due to poor sanitary conditions and a general health decline due to exposure to extreme weather [30]. In addition, rough sleepers face barriers to accessing the healthcare system due to the lack of a permanent address, lack of health insurance, or social deprivation [31].

Access to, as well as the state of, the healthcare system has a profound effect on prevention and treatment of health conditions [32]. A good healthcare system needs to be accessible in terms of proximity, as people need to be able to access primary care easily [33]. In addition, healthcare needs to be accessible in terms of admission and waiting times [34]. Financial factors also determine the health status of people, as many poor individuals have no supplementary healthcare access other than the one freely offered, as well as to nonprescribed medicine [9,10]. Ultimately, access to healthcare or to community assets, in general, depends on other factors such as personal safety; the detrimental health effects from exposure to crime and violence, for instance, include hypertension, respiratory, cardiovascular, and mental health problems [35].

The current study quantified the effects of community assets on major health conditions using a spatiotemporal data set covering the population of England at a fine spatial scale over six years. Community assets included green space, environmental conditions, health system performance, the built environment, and financial variables [9,10,19,36,37,38], depending on the data available. Rather than formulate any explicit hypotheses, a data-driven analysis was performed, making the implicit hypothesis that an underlying interdependence among the collected data can be objectively mined [39,40]. Data-driven approaches are not in conflict with hypothesis-led studies in scientific knowledge discovery but are complementary and iterative with them [41,42].

In order to ask the question ‘What are the effects of community assets on major health conditions in England?’, the research employed a data analytic approach, as follows: Publicly available spatiotemporal data at the level of the local healthcare unit were mined in terms of health conditions and community assets. Data were standardised sequentially to facilitate comparisons in space and time across differing demographics. Cluster analysis of health conditions and community assets was performed. Using each health condition as a dependent variable, the effects of community assets were quantified using statistical models. Model selection was performed by eliminating the least informative community assets per health condition. The diversity of the remaining community assets as significant predictors of a health condition was quantified. The percentage of unique variance explained by each community asset on each health condition was assessed using hierarchical variance partitioning within an analysis of variance model. The results are synthesised and discussed.

## 2. Methods

### 2.1. Study Area and Data

**Figure 1:**
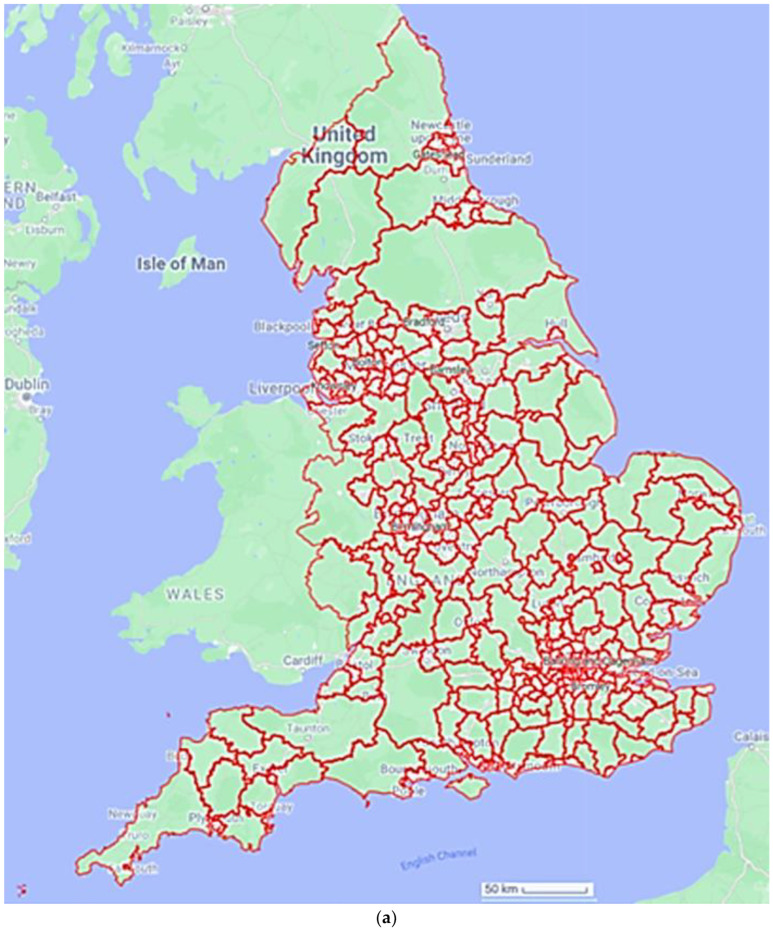

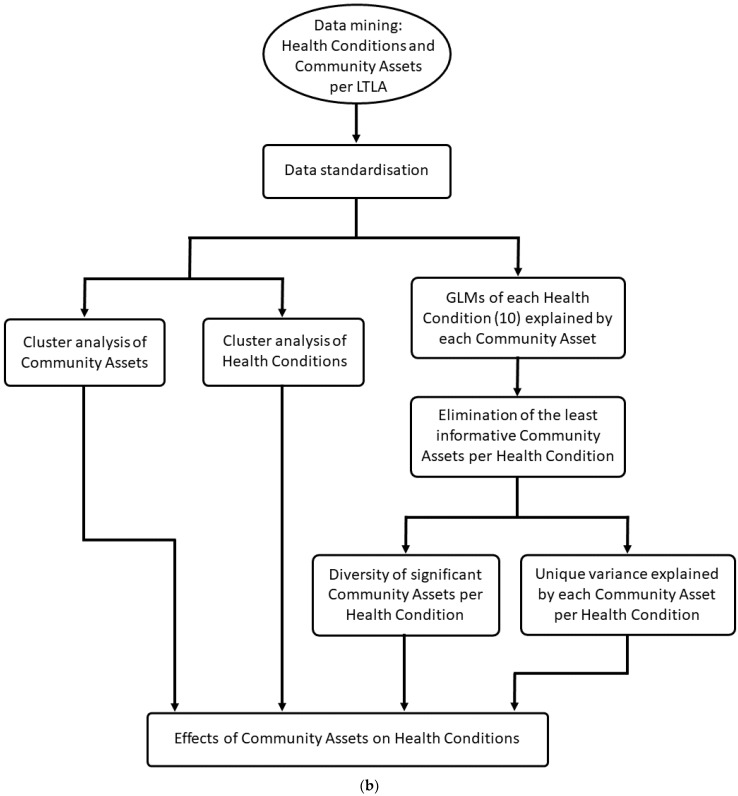
(**a**) A spatial plot of a local healthcare unit (LTLA). Map plotted in Google maps using data from the Geoportal Statistics UK, available at: https://geoportal.statistics.gov.uk/datasets/196d1a072aaa4882a50be333679d4f63/explore?showTable=true (accessed 20 February 2024). (**b**) Block diagram of the framework applied here. Initially data were mined from publicly available spatiotemporal data sets at the level of an LTLA. Data were standardised sequentially to facilitate comparisons across space, time, and unequal demographics. Clusters of community assets and health conditions were computed and visualised. Generalised linear models (GLMs) were fitted for each health condition as dependent variables and community assets as explanatory variables. Model selection was performed for each GLM eliminating the least informative community asset variables per health condition. The diversity of community assets as significant predictors per health condition was calculated. Hierarchical variance partitioning between each health condition and the significant explanatory community assets was computed indicating the unique variance explained by each community assets per health condition.

The data on major health conditions and community assets were retrieved from publicly available databases of the NHS-registered population of England, 57.1 million people. The data included ten indices of major health conditions and ten indices of community assets (Table 1). The temporal replicate encompassed six years (2015–2020) at an annual temporal resolution. The spatial extent covered England at a spatial resolution of lower tier local authority (LTLA) with a spatial replicate of 308 LTLAs per annum (Figure 1a). For a block diagram of the proposed approach, see Figure 1b. Data are available at: https://www.ons.gov.uk/peoplepopulationandcommunity/healthandsocialcare/healthandwellbeing/articles/howhealthhaschangedinyourlocalarea2015to2020/2022-11-09 (accessed on 20 February 2024) and https://www.ons.gov.uk/economy/environmentalaccounts/datasets/accesstogardensandpublicgreenspaceingreatbritain) (accessed on 20 February 2024).

The health conditions examined here account for over 60% of the years lost to early death or lived in ill health in England [3]. Thus, the data set used here has a substantial temporal replicate, a fine spatial resolution allowing for detailed differentiations among locations and covering a large number of individuals across the major health conditions. In addition, the data set is open access, facilitating transparency and accountability. More detailed data sets exist for specific locations or health conditions but do not permit comparisons in other locations or health conditions due to their limitations or data format. Data from later years were still not available for all locations and health conditions and using some of them would have compromised both the spatial extent and the number of health conditions and community assets examined. In addition, data from 2021 and onwards include COVID-19 cases, with additional interactions with the major health conditions examined here. Our intention was not to map the most recent situation in the local healthcare units; instead, we sought to quantify complex relationships between health conditions and community assets.

### 2.2. Health Conditions

High blood pressure was calculated as the weighted number of people answering ‘yes’ to ‘high blood pressure’ in the question, ‘Which, if any, of the following long-term conditions do you have?’, divided by the total number of surveys. Source: GP patient survey, NHS statistics.

Obesity was calculated as the number of adults aged 18+ with a body mass index (BMI) classified as overweight (including obese) divided by the number of adults aged 18+ with a valid height and weight recorded. Source: Fingertips (93088).

Dementia was calculated as the weighted percentage of the number of people answering ‘yes’ to ‘Alzheimer’s disease or other cause of dementia’ in the question, ‘Which, if any, of the following long-term conditions do you have?’, divided by the total number of surveys. Source: GP patient survey, NHS statistics.

Diabetes was calculated as the weighted percentage of the number of people answering ‘yes’ to ‘diabetes’ in the question, ‘Which, if any, of the following long-term conditions do you have?’, divided by the total number of surveys. Source: GP patient survey, NHS statistics.

Mental health was calculated as the weighted percentage of the number of people answering ‘yes; to ‘a mental health condition’ in the question ‘Which, if any, of the following long-term conditions do you have?’, divided by the total number of surveys. Source: GP patient survey, NHS Statistics.

Cardiovascular conditions were calculated as the weighted percentage of the number of people answering ‘yes’ to ‘heart condition, such as angina or atrial fibrillation’ in the question, ‘Which, if any, of the following long-term conditions do you have?’, divided by the total number of surveys. Source: GP patient survey, NHS statistics.

Musculoskeletal conditions were calculated as the weighted percentage of the number of people answering ‘yes’ to ‘arthritis or ongoing problem with back or joints’ in the question, ‘Which, if any, of the following long-term conditions do you have?’, divided by the total number of surveys. Source: GP patient survey, NHS statistics.

Respiratory conditions were calculated as the weighted percentage of the number of people answering ‘yes’ to ‘breathing condition such as asthma or COPD’ in the question, ‘Which, if any, of the following long-term conditions do you have?’, divided by the total number of surveys. Source: GP patient survey, NHS statistics.

Kidney and liver disease was calculated as the weighted percentage of the number of people answering ‘yes’ to ‘kidney or liver disease’ in the question, ‘Which, if any, of the following long-term conditions do you have?’, divided by the total number of surveys. Source: GP patient survey, NHS statistics.

Cancer was calculated as the weighted percentage of the number of people answering ‘yes’ to ‘self-reported cancer (diagnosis or treatment in the last 5 years)’ in the question, ‘Which, if any, of the following long-term conditions do you have?’, divided by the total number of surveys. Source: GP patient survey, NHS Statistics.

### 2.3. Community Assets

Community assets were considered in a broad sense here, including social infrastructure, green space, clean air, access to healthcare, and living standards. Whilst a community asset has a positive effect, a pragmatic approach was used, which also included inverse indicators for practical reasons related to data availability and interoperability across the study area and the time span. Community asset variables included distance to parks and access to private outdoor space as indices of green space. Distance to a general practitioner (GP) service and acceptable GP appointment times were selected as indices of the health system. Distance to sport and leisure facilities was selected as a proxy of public access to health and wellbeing activities. Air pollution was selected as an inverse proxy of cleanliness of the environment, personal crime for safety of the living space and location, and household overcrowding on the partitioning of living space. Absolute poverty, indicated by the number of children in low-income families, as well as rough sleeping (i.e., homelessness) were selected as indicators of financial deprivation.

Distance to a park, public garden, or playing field (‘parks’) was calculated as the median distance in km from all addresses (houses and flats) to the nearest park. Source: calculated by the Office of National Statistics (ONS) using data from Natural England and postcode centroids from the ONS Geoportal website.

Access to private outdoor space was calculated as a weighted percentage of the number of addresses (houses and flats) with private outdoor space divided by the total number of addresses in each LTLA. Source: produced by ONS using ONS and Ordnance Survey data, available at the ONS website.

Distance to GP services (primary care distance) was calculated as a median distance in km of all addresses to the nearest GP practice services. Source: calculated by ONS using GP practice addresses form NHS digital and postcode centroids from the ONS Geoportal website.

GP appointments (primary care waiting times) was calculated as the weighted percentage of people answering ‘No’ and ‘I did not take an appointment’ to ‘Were you satisfied with the type of appointment (or appointments) you were offered?’, divided by the total number of surveys. Source: GP patient survey NHS Statistics.

Distance to sport and leisure facilities was calculated as the median distance in km from all addresses (houses and flats) to the nearest sport facility address. Source: calculated by ONS using sport facility addresses from Sport England (Sport Facility addresses from Active Places Power website) and postcode centroids from the NSPL (ONS Geoportal website).

Air pollution was calculated as population-weighted annual mean PM2.5 in µg m^−3^. Source: Defra website, UK air.

Personal crime was calculated as the sum of personal crime offenses (violence against the person, sexual offences, robbery, theft, criminal damage, and arson) per 1000 persons, mid-year population estimates. Source: ONS website, recorded crime data by Community Safety Partnership area. ONS website for population estimates for denominator.

Household overcrowding was calculated as the weighted percentage of the sum of number of households with occupancy rating of −1 and −2 or less, divided by the number of households. Occupancy rating provides a measure of whether a household’s accommodation is overcrowded or under-occupied. An occupancy rating of negative 1 or less implies that a household has fewer bedrooms than required according to the Bedroom Standard, so is overcrowded (for example, negative 1 means one bedroom fewer than required, negative 2 has two fewer than required). Source: Nomis (QS408EW).

Absolute poverty was calculated as the weighted percentage of the number of children aged under 16 years living in absolute low-income families, divided by the total number of children. Source: Department for Work and Pensions, gov.uk website: children in low-income families.

Rough sleeping was calculated as the weighted percentage of people sleeping rough on a single night between 1st October and 30th November per 100,000 residents of the area. Source: Ministry of Housing, Communities and local government, gov.uk website: rough sleeping statistics. ONS website for population estimates for denominator.

### 2.4. Data Standardisation

Demographic factors play important roles in health conditions and need to be accounted for when evaluating the effects of community assets across time and space [43]. In order to facilitate comparisons among LTLAs with substantially different populations, health conditions, and community assets, data were standardised [43]. Variables for each health condition and community asset were scaled to obtain a mean value of 100 and a standard deviation of 10, with 2015 as the base year. For example, obesity was scaled using the mean obesity value for England in 2015. Values higher than 100 indicate locations or years within the same location with less obesity than the mean of England in 2015, and values below 100 indicate worse. The scale is such that for indicators at the LTLA level, a score of 110 represents a score of one standard deviation higher than England’s 2015 score for that same variable, and 120 represents a value two standard deviations higher, etc. This process ensures that all variables are spatiotemporally comparable and that assumptions of the analysis of variance (ANOVA) in terms of residual heteroscedasticity are successfully met. All analyses were conducted in R open access statistical software [44].

### 2.5. Cluster Analysis of Health Conditions and Community Assets

In order to quantify correlations and clustering groups among variables, cluster analysis was performed for all health conditions’ variables [45]. The cluster analysis was replicated for community asset variables. The cluster analysis deployed a hierarchical procedure to form the clusters [45,46]. Variables were grouped together that were correlated (similarity) with each other [45,47]. At each step, two clusters were joined, until just a single cluster was formed at the final step. Similarity and distance values were calculated for the clusters at each step to determine the final grouping of variables [47]. Cluster analysis can be used to detect meaningful subgroups in a sample [48]. Clusters were calculated using complete linkage, one of several methods of agglomerative hierarchical clustering. Complete linkage clustering considers the distance between two clusters to be the distance between their most distant data points. The method can result in more compact, evenly sized clusters compared to single linkage. It is less sensitive to noise and outliers, as it minimises the maximum distance between the points in each cluster using Pearson correlation. It is suited to cases in which it is important to ensure that clusters are composed of closely related points, such as the data used here deriving from potentially unequal and distant LTLAs. The final cluster, which can be visualised as a dendrogram consists, of a single cluster with subclusters grouped by similarity levels [49].

### 2.6. Effects of Community Assets on Health Conditions

Generalised linear models (GLMs) were fitted for each health condition as a dependent variable [50,51]; ten models were fitted in total, one for each health condition. Independent explanatory variables included all ten community asset variables. Model selection of the most parsimonious model structure eliminating the noninformative community asset variables was performed for each health condition using the Akaike information criterion (AIC) [52]. Any deletion of nonsignificant variables that did not increase AIC > 2 was deemed justified [53]. The variables that remained in the final most parsimonious (i.e., optimal) model, after model selection, were recorded with their effect sizes (i.e., model coefficients) thereby quantifying the identity and diversity of the community assets that were significant predictors for each health condition.

### 2.7. Variance Partitioning per Community Asset

Quantifying the variance uniquely explained by multiple variables is a powerful computational tool for understanding the explanatory power of each variable, especially when variables are correlated. Given that ANOVA evaluates whether variance among groups is greater than the variance within a group, the test can be used to partition variance by dividing the total variance into the sources or predictors of that variation. Hierarchical variance partitioning additionally describes the relative importance of individual predictors or groups of predictors. Hierarchical variance partitioning was performed among the independent variables of the optimal GLM for each health condition to account for the unique contribution of each explanatory variable to the total variance of that health condition [54]. Variance partitioning is a computational statistical technique capable of handling potentially correlated independent variables, whilst ranking the predictor importance of each variable [54,55,56]. It is calculated from the AIC weights of each independent variable and based upon the number of times that a variable was significant among all possible combinations of the explanatory variables [55]. The ‘average shared variance’ method for the predictor in multiple regression and canonical analyses was used [57]. This method suggests that shared variance can be partitioned into equal components according to the number of predictors involved, so that the relative importance of each predictor can be estimated by its part *R*^2^ plus the sum of all allocated average shared *R*^2^ and unique variance attributed to each variable can be quantified [57]. Whilst the sum of the relative importance of each predictor in terms of the explained variance adds to 100%, the unique variance explained by each predictor is smaller than or equal to 100%. Here, the amount of unique variance was computed to explain the contribution by each significant community asset as a predictor of a health condition.

## 3. Results

### 3.1. Clusters

**Figure 2:**
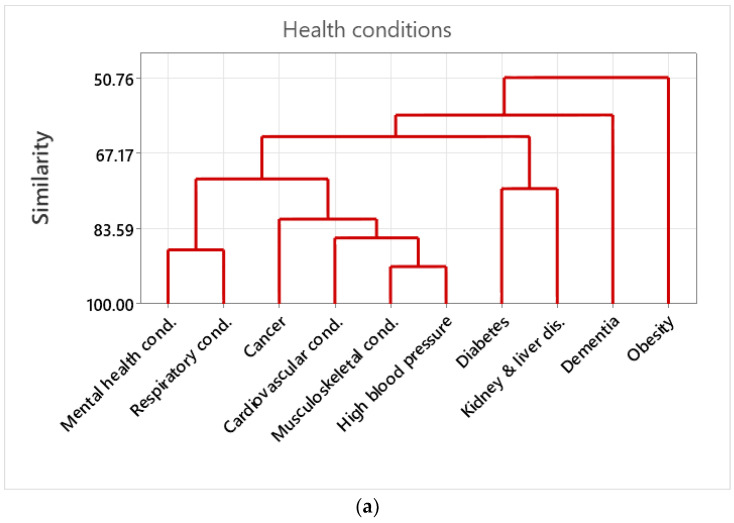

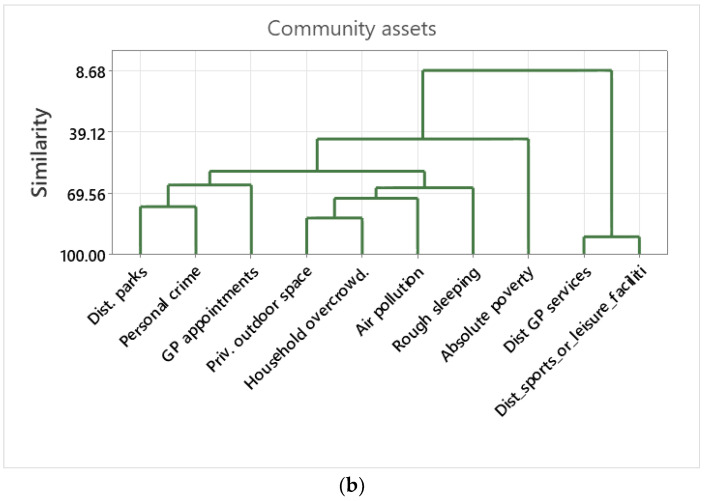
(**a**) Dendrograms of the cluster analysis among health conditions. The cluster analysis deploys a hierarchical procedure to form the clusters. Variables were grouped together that are correlated (i.e., similarity) to each other. Similarity is indicated by the Pearsons’ correlation values. (**b**) Dendrograms of the cluster analysis among the assets. The cluster analysis deployed a hierarchical procedure to form the clusters. Variables were grouped together that are correlated (i.e., similarity) with each other. Similarity is indicated by the Pearsons’ correlation values.

Cluster analysis of health conditions indicated that musculoskeletal conditions had the highest similarity with high blood pressure, forming a larger cluster together with cardiovascular conditions and cancer. Mental health conditions exhibited high similarity with respiratory conditions forming a cluster. Diabetes and kidney and liver disease formed a cluster together, having lower similarities than other clusters, whilst dementia and obesity did not cluster well together with other major health conditions (Figure 2a).

The cluster analysis of community assets indicated that distance to sports or leisure facilities had very high similarity with distance to GP services. Private outdoor space had high similarity with household overcrowding, forming a larger cluster together with air pollution and rough sleeping. Distance to parks and personal crime had high similarity, forming a larger cluster with GP waiting times. Absolute poverty was isolated from other community asset variables (Figure 2b).

### 3.2. Effects of Community Assets on Health Conditions

The optimal model between mental health conditions and community assets included the effects of distance to parks, private outdoor space, distance to GP services, GP waiting times, air pollution, household overcrowding, personal crime, absolute poverty, and rough sleeping and was calculated using ANOVA (Table 2).

The optimal model between high blood pressure and community assets included the effects of distance to parks, private outdoor space, distance to GP services, distance to sports or leisure facilities, GP waiting times, air pollution, personal crime, and rough sleeping and was calculated using ANOVA (Table 3).

The optimal model between obesity and community assets included the effects of distance to parks, private outdoor space, GP waiting times, distance to sports or leisure facilities, household overcrowding, personal crime, and absolute poverty and was calculated using ANOVA (Table 4).

The optimal model between cancer and community assets included the effects of private outdoor space, distance to GP services, GP waiting times, distance to sport or leisure facilities, air pollution, absolute poverty, and rough sleeping and was calculated using ANOVA (Table 5).

The optimal model among cardiovascular conditions included the effects of distance to parks, private outdoor space, distance to GP services, GP waiting times, air pollution, household overcrowding, absolute poverty, and rough sleeping and was calculated using ANOVA (Table 6).

The optimal model between diabetes and community assets included the effects of distance to parks, private outdoor space, distance to GP services, GP waiting times, distance to sports or leisure facilities, household overcrowding, personal crime, absolute poverty, and rough sleeping and was calculated using ANOVA (Table 7).

The optimal model between dementia and community assets included the effects of distance to parks, GP waiting times, distance to sports or leisure facilities, air pollution, personal crime, and absolute poverty and was calculated using ANOVA (Table 8).

The optimal model between kidney and liver disease and community assets included the effects of distance to parks, private outdoor space, GP waiting times, air pollution, absolute poverty, and rough sleeping and was calculated using ANOVA (Table 9).

The optimal model between musculoskeletal conditions and community assets included the effects of distance to parks, private outdoor space, distance to GP services, GP waiting times, air pollution, household overcrowding, absolute poverty, and rough sleeping and was calculated using ANOVA (Table 10).

The optimal model between respiratory conditions and community assets included the effects of distance to parks, distance to GP services, GP waiting times, air pollution, household overcrowding, personal crime, and rough sleeping and was calculated using ANOVA (Table 11).

### 3.3. Diversity of Community Assets and Health Conditions

**Figure 3:**
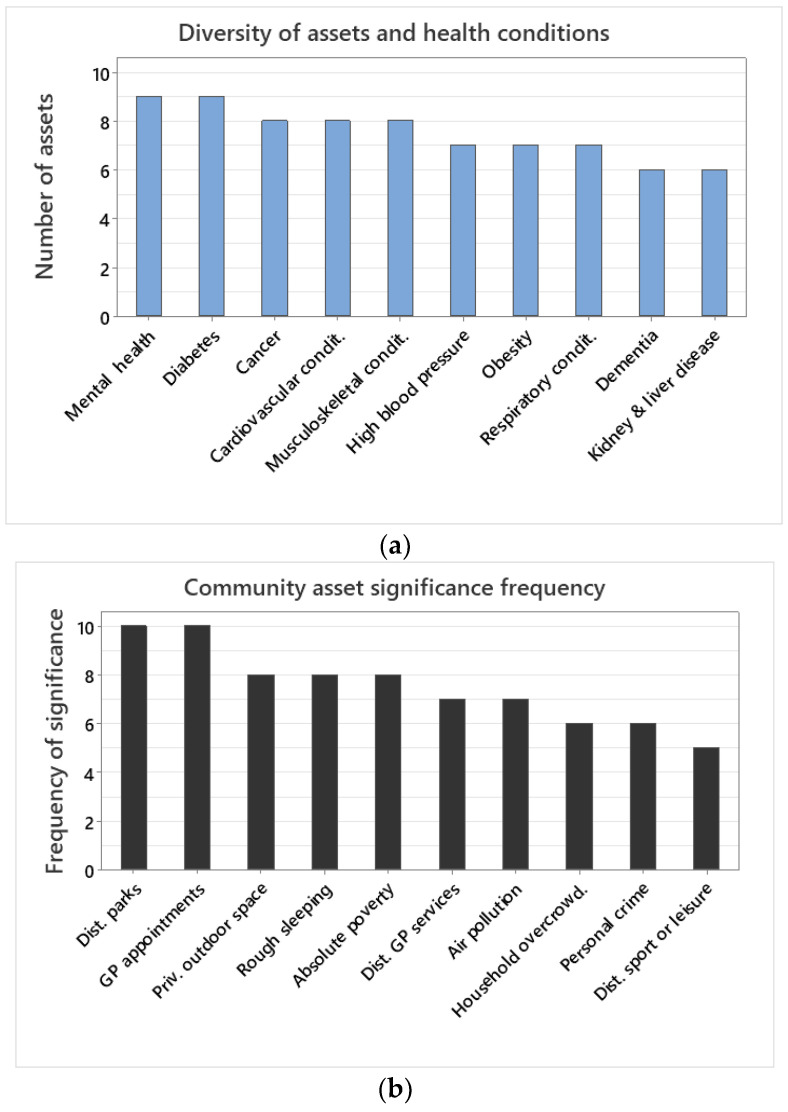
(**a**) Number of community assets included in the final model between a health condition (i.e., dependent variable) and the ten community asset explanatory variables investigated. The final model refers to the community assets included in the model after model selection eliminating the least informative ones. (**b**) Number of times that a community asset was included in the final model for a health condition.

Among health conditions, mental health, and diabetes were the most complex, each meriting nine community asset predictor variables (Figure 3a). Cancer, cardiovascular, and musculoskeletal conditions were best explained by eight community asset variables (Figure 3a). High blood pressure, obesity, and respiratory conditions were best explained by seven community asset variables (Figure 3a). Dementia and kidney and liver disease were best explained by six community asset variables (Figure 3a).

### 3.4. Community Asset Significance Frequency

Among community assets, GP waiting times and distance to parks were always significant predictors for all health conditions (Figure 3b). Private outdoor space, rough sleeping, and absolute poverty were significant in eight out of ten health conditions (Figure 3b). Distance to GP services and air pollution were significant predictors of seven health conditions (Figure 3b). Household overcrowding and personal crime were significant predictors of six, whilst distance to sports or leisure facilities were of five health conditions (Figure 3b).

### 3.5. Variance Explained per Community Asset

**Figure 4:**
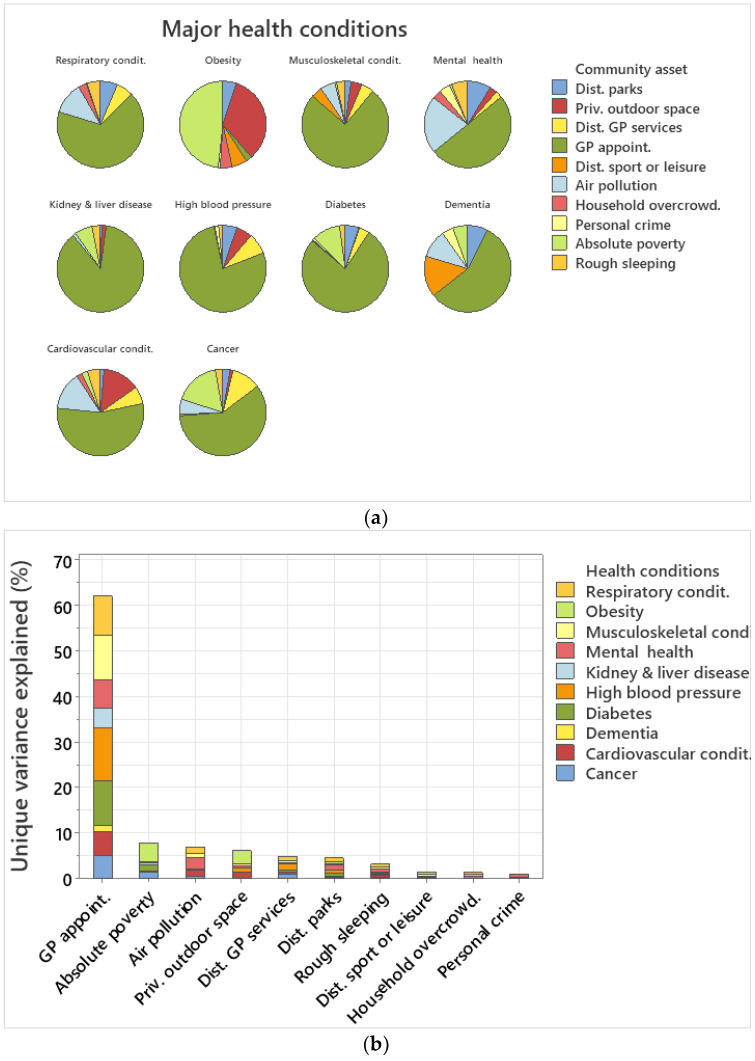

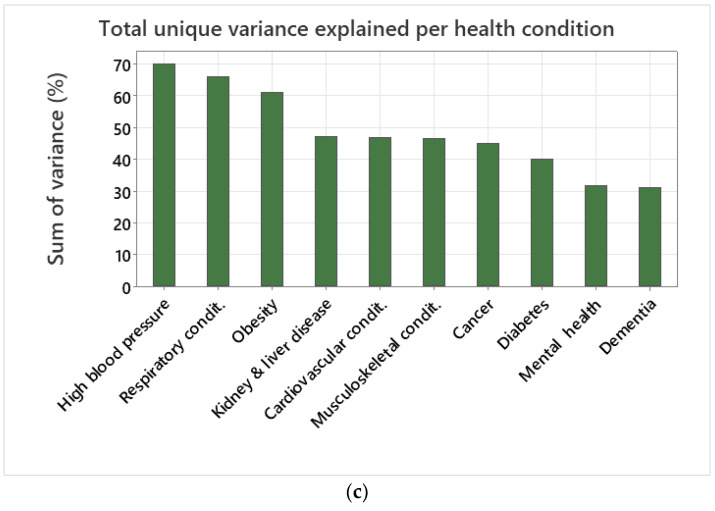
(**a**) Pie chart of unique variance explained by each community asset per health condition. (**b**) Sum of total unique variance explained by each community asset across health conditions. (**c**) Sum of total unique variance explained per health condition.

In terms of unique variance explained by each community asset for each health condition, GP waiting time was the best explanatory covariate for nine out of ten health conditions explaining >50% of unique variance in each health condition (Figure 4a). GP waiting times explained 77% of the variance of diabetes, 86.9% of kidney and liver disease, 77.6% of high blood pressure, 75.4% of musculoskeletal, and 67% of respiratory conditions (Figure 4a). Absolute poverty explained 48.3% and private outdoor space 33.5% of unique variance for obesity, the only health condition that GP waiting times did not explain the highest percentage of variance (Figure 3a). Air pollution explained 21.4% of variance regarding mental health conditions, 14.3% of cardiovascular conditions, and 5.7% of cancer (Figure 4a). Private outdoor space explained 33.5% of the variance for obesity and 13.8% for cardiovascular conditions (Figure 4a). Distance to GP services explained 10.8% of the variance for cancer whilst distance to sport or leisure facilities 15.3% of variance for dementia (Figure 4a). Distance to parks explained 8.7% of the unique variance for mental health, 7.5% of dementia, 5.3% of high blood pressure, and 5.1% of obesity (Figure 4a).

In terms of unique variance explained cumulatively per community asset across all ten health conditions, GP waiting times explained 62.15%, absolute poverty 7.95%, air pollution 7%, private outdoor space 6.21%, distance to GP services 4.9%, distance to parks 4.6%, rough sleeping 3.2%, distance to sport facilities 1.55%, household overcrowding 1.4%, and personal crime 0.99% (Figure 4b).

In terms of unique variance explained per health condition, the best explained health condition variance was for high blood pressure (69.84%), followed by respiratory conditions (65.87%), obesity (60.98%), kidney and liver disease (47.10%), cardiovascular conditions (46.74%), musculoskeletal conditions (46.52%), cancer (44.84%), and diabetes (39.89%) (Figure 4c). Health conditions with a low percentage of variance explained included mental health (31.62%) and dementia (30.95%) (Figure 4c).

## 4. Discussion

The major contribution of the data analytic approach performed here indicates that communities with high diversity and richness of assets [19,58] are able to perform better in terms of addressing major health conditions. A novel finding from the research was that mental health and diabetes required all ten community assets examined here, whilst cancer, cardiovascular and musculoskeletal conditions required nine community assets, indicating that complex health conditions require a large number of assets [59,60,61,62,63]. The community assets used here exhibited high significance across health conditions and explained the high levels of unique variance for each health condition. Mental health, dementia, and diabetes were the least explained health conditions in terms of total variance by the community assets included, indicating that additional variables regarding behavioural aspects and quality of life may increase the explanatory power of these health conditions [64,65,66]. Whilst all community assets may be important for building an asset-diverse community, a further contribution of the research was the finding that GP waiting times and distance to public green space and parks were always significant predictors for all of the health conditions examined. Thus, these factors should be immediate priority in terms of assets and interventions, and their mapping and improvement may result in the amelioration of major health conditions [67,68].

GP waiting times explained the vast majority of variance, both cumulatively and individually, across health conditions. GP appointments are the key entry point into the health system, and early access is key to an early diagnosis and prevention. Overall, when a major condition is identified early, the outcome is much better and the impact on a person’s life much reduced [3]. Long GP waiting times present a significant barrier to healthcare access for a range of health services [34]. There is also evidence that waiting time is unequally distributed against those of lower socioeconomic status [34,69]. Improving GP waiting times should be a priority for the government in the UK, as the results indicate that decreasing waiting times will have a vast impact across health conditions. Furthermore, this improvement is highly likely to act on relatively short time scales. Other studies have noted the importance of GP waiting times and suggest interventions for improvement [70].

The significance of public green space in terms of distance to parks was recorded across all health conditions examined. In particular, public green space was very important in terms of the variance explained for mental health conditions, one of the most complex and least predictable health conditions in terms of the number of assets required, in addition to the total variance explained by community assets for this health condition. Distance to parks was also important for explaining the variance of respiratory conditions [71,72], cardiovascular conditions [73,74], and dementia [75,76], as well as health conditions in general, as reported in other studies [77,78,79]. Private green space was clustered with household overcrowding, indicating few privileged people having access to it in high population density areas.

Community assets, as well as health conditions, were correlated, as exemplified by the cluster analysis. Improvements in community assets are likely to be more pronounced in health conditions in which their variance was best explained, such as high blood pressure, respiratory conditions, and obesity. Distance to parks was strongly clustered with personal crime, and, therefore, LTLAs with a long distance to the nearest park have high levels of personal crime. In addition, GP waiting times were also clustered with the cluster distance to parks and personal crime with a high similarity indicating that GP waiting times are higher at LTLAs without nearby green space and high levels of personal crime. Apart from the effect that personal crime has on a person’s health and wellbeing per se, it also acts as a barrier to accessing green space and health services [80]. Thus, improvement in GP waiting times and access to public green space should be combined with safety improvements.

Regarding other health conditions, there is a four-way cluster between high blood pressure and musculoskeletal conditions clustered with cardiovascular conditions clustered with cancer. This cluster is linked with a second strong clustering between mental health and respiratory conditions. In England, people with two or more conditions account for 50% of hospital admissions and primary care visits, and >50% of NHS costs [3]. In addition, 92% of people with cardiovascular conditions, and 70% of those with mental health issues have at least one other condition [3,81]. Therefore, focusing on these clusters will benefit multiple health conditions. Apart from GP waiting times and access to green space, cardiovascular and respiratory conditions are explained by air pollution and, thus, cleaner air would facilitate their improvement [82,83]. In terms of costs, diabetes accounts for 10% of the annual NHS budget [81]. A large fraction of the diabetes prevalence is explained by absolute poverty, and, to that end, more supportive financial assistance to poor families or for healthy meals would reduce the burden on the health system [84]. In addition, mental health and respiratory conditions have a high similarity. Other studies have reported that a person may be more likely to experience poor mental wellbeing or a mental health condition if living with a respiratory condition [85], possibly due to difficulties in carrying out activities compared with previously, breathing anxiety, or the frustration of needing regular medical treatment [86].

### Limitations and Future Research

The effect of public green space, as quantified here, is likely to be underestimated in terms of the variance explained as the introduction of new parks, and, thus, changes in the distance to the nearest park across the spatial resolution, typically takes longer than the six years examined here. Thus, there is low differentiation of the distance to parks over time, and the effect of public green space is quantified by the spatial differentiation among locations, but the temporal differentiation within the same location is low and may result in an underestimation of the variance explained [87]. Another limitation is the appropriate size of a community for this type of study; whilst health and geoportal statistics can be obtained for any size of area, it does not mean that the same conclusions can necessarily be applied to other geographic locations of varying sizes. Furthermore, inaccuracies due to loss of information can arise in modelling, as it cannot be assumed that data typically collected at different levels of spatial aggregation can be applied to arbitrary sizes of area. This study focused on community assets and used GP waiting times [88,89] and distance to GP services [90,91,92] as indices of healthcare system. However, there are several other healthcare system performance indicators [93,94] not investigated here. The analysis did not include genetic factors that are also explanatory covariates of major health conditions [9] and which could be considered in future research. The multiple health conditions examined here refer to the characteristics of individuals of the primary care unit area examined (LTLA) and not of individuals per se. Some variables, like personal crime and child poverty, are significant predictors for many health outcomes, such as high blood pressure. It is likely that rather than showing a direct cause and effect relationship, these variables have a correlational relationship where other, possibly unobserved, factors may contribute to the association with the health outcomes. The community assets deployed here as explanatory variables of major health conditions may, for example, act as indirect covariates for socioeconomic status, not accounted for here.

## 5. Conclusions

Public health outcomes can depend on a wide range of health determinants including community assets. It is imperative to understand the role of localised services in relieving some of the pressures of multiple comorbid conditions on health systems. The quantification of the effect of community assets on major health conditions outlined within this paper highlights the importance of such localised amenities and services to multiple comorbidities. Environments such as green space, pollution, poverty, the urban environment, safety, and sport and leisure facilities were related to high blood pressure, obesity, dementia, diabetes, mental health, cardiovascular conditions, musculoskeletal conditions, respiratory conditions, kidney and liver disease, and cancer. Both major health conditions and community assets were often clustered, with the results suggesting that the diversity and richness of community assets are key to major health condition outcomes.

The analysis of the broader determinants of health quantified here indicated that most were accounted for by healthcare system performance and distance to public green space, whilst socioeconomic factors were also important. Emphasising community approaches, quantifying significant relationships, and understanding asset strengths and deficits are needed to address these broader determinants followed by targeted interventions [95]. Whilst the performance of the public health system remains of key importance, emphasis should be sought to strengthen different assets and services locally [96]. Data analytic approaches such as the one performed here can handle large data sets, including large numbers of individuals, incidents, health conditions, locations, temporal replication, and their complex relationships. Data analytic approaches to analysing these factors can, thereby, help determine the unique variance explained by each community asset in order to understand the bigger picture, though they may need to be combined with finer-scale studies and qualitative surveys to examine the effects of specific geographic locations, genetic factors, and socioeconomic status. Emphases on community approaches, significant relationships, and asset strengths and deficits are needed alongside targeted interventions. Whilst the performance of the public health system remains of key importance, community assets and local infrastructure remain paramount to the broader determinants of health.

## Figures and Tables

**Table 1:** Health condition and community asset indices and their units. Polarity refers to whether lower or higher scores are desirable. In order to facilitate comparisons among locations with substantially different populations, health conditions and community assets, data were standardised and, thus, used as % scores (see Section 2.4 Data standardisation).

Variable	Type of Value and Unit	Polarity
**Health condition:**		
Mental health conditions	%	Low is good
Cancer	%	Low is good
Cardiovascular conditions	%	Low is good
Dementia	%	Low is good
Diabetes	%	Low is good
Kidney and liver disease	%	Low is good
Musculoskeletal conditions	%	Low is good
Respiratory conditions	%	Low is good
High blood pressure	%	Low is good
Obesity and overweight in adults	%	Low is good
**Community asset:**		
Private outdoor space	%	High is good
Distance to GP services	Median, km	Low is good
Distance to sports or leisure facilities	Median, km	Low is good
GP waiting times	%	Low is good
Personal crime	Per 1000 persons	Low is good
Absolute poverty	%	Low is good
Air pollution	Population-weighted annual mean PM2.5 in µg m^−3^	Low is good
Household overcrowding	%	Low is good
Road safety	Accidents per km^2^	Low is good
Rough sleeping	Per 100,000	Low is good
Distance to parks	Median, km	Low is good

**Table 2:** ANOVA results of the optimal model for mental health conditions. The nonsignificant effects of private outdoor space cannot be eliminated based on the AIC and, thus, remain in the final model after model selection. Variables with *p*-values < 0.001 are indicated with ***, variables with *p*-values < 0.01 with **, and variables with *p*-values < 0.05 with *. Final model AIC = 12,722, dAIC = 1.68.

	Degrees of Freedom (df)	Deviance	Residual df	Residual Deviance	Pr(>Chi)	Sig.
NULL			1841	161,239		
Dist_parks	1	6552.4	1840	154,687	<0.001	***
Private_outdoor_space	1	19.2	1839	154,668	0.566	
Dist_GP_services	1	1662	1838	153,006	<0.001	***
Acceptable_GP_appointment	1	28,939.6	1837	124,066	<0.001	***
Air_pollution	1	10,539	1836	113,527	<0.001	***
Household_overcrowding	1	298.1	1835	113,229	0.024	*
Personal_crime	1	3011.9	1834	110,217	<0.001	***
Child_poverty	1	628.9	1833	109,588	0.001	**
Rough_sleeping	1	3116.1	1832	106,472	<0.001	***

**Table 3:** ANOVA results of the optimal model for high blood pressure. The nonsignificant effects of distance to parks cannot be eliminated based on the AIC and, thus, remained in the final model after model selection. Variables with *p*-values < 0.001 are indicated with ***, variables with *p*-values < 0.01 with **. Final model AIC = 13,115, dAIC = 2.

	Degrees of Freedom (df)	Deviance	Residual df	Residual Deviance	Pr(>Chi)	Sig.
NULL			1841	222,890		
Dist_parks	1	2	1840	222,887	0.858	
Private_outdoor_space	1	8329	1839	214,558	<0.001	***
Dist_GP_services	1	10,638	1838	203,920	<0.001	***
Dist_sports_or_leisure_facilities	1	830	1837	203,091	<0.001	***
Acceptable_GP_appointments	1	69,044	1836	134,047	<0.001	***
Personal_crime	1	727	1835	133,320	0.002	**
Rough_sleeping	1	1414	1834	131,906	<0.001	***

**Table 4:** ANOVA results for the optimal model for obesity. The nonsignificant effects of household overcrowding cannot be eliminated based on the AIC and, thus, remained in the final model after model selection. Variables with *p*-values << 0.001 are indicated with ***, and variables with *p*-values < 0.05 with *. Final model AIC = 12,573, dAIC = 1.824.

	Degrees of Freedom (df)	Deviance	Residual df	Residual Deviance	Pr(>Chi)	Sig.
NULL			1841	200,352		
Dist_parks	1	292	1840	200,060	0.02	*
Private_outdoor_space	1	64,298	1839	135,762	<0.001	***
Acceptable_GP_appointments	1	4153	1838	131,609	<0.001	***
Dist_sports_or_leisure_facilities	1	210	1837	131,398	0.048	*
Household_overcrowding	1	4	1836	131,394	0.784	
Personal_crime	1	10,971	1835	120,423	<0.001	***
Child_poverty	1	22,056	1834	98,367	<0.001	***

**Table 5:** ANOVA results for the optimal model for cancer. The nonsignificant effects of distance to sports of leisure facilities cannot be eliminated based on the AIC and, thus, remained in the final model after model selection. Variables with *p*-values < 0.001 are indicated with ***, variables with *p*-values < 0.05 with *. Final model AIC = 13,085, dAIC = 1.58.

	Degrees of Freedom (df)	Deviance	Residual df	Residual Deviance	Pr(>Chi)	Sig.
NULL			1841	181,694		
Dist_parks	1	57,16.4	1840	175,977	<0.001	***
Private_outdoor_space	1	2100.2	1839	173,877	<0.001	***
Dist_GP_services	1	15,118.7	1838	158,758	<0.001	***
Acceptable_GP_appointments	1	20,817.2	1837	137,941	<0.001	***
Dist_sports_or_leisure_facilities	1	65.2	1836	137,876	0.337	
Air_pollution	1	454.1	1835	137,422	0.011	*
Child_poverty	1	6586	1834	130,836	<0.001	***
Rough_sleeping	1	1152.3	1833	129,684	<0.001	***

**Table 6:** ANOVA results for the optimal model for cardiovascular conditions. Variables with *p*-values < 0.001 are indicated with ***, variables with *p*-values < 0.01 with **. Final model AIC = 12,650, dAIC = 1.87.

	Degrees of Freedom (df)	Deviance	Residual df	Residual Deviance	Pr(>Chi)	Sig.
NULL			1841	170,547		
Dist_parks	1	8287.1	1840	162,260	<0.001	***
Private_outdoor_space	1	23,954.1	1839	138,306	<0.001	***
Dist_GP_services	1	6393.1	1838	131,913	<0.001	***
Acceptable_GP_appointments	1	21,357.2	1837	110,556	<0.001	***
Air_pollution	1	4637.9	1836	105,918	<0.001	***
Household_overcrowding	1	447.7	1835	105,470	0.005	**
Child_poverty	1	902.6	1834	104,568	<0.001	***
Rough_sleeping	1	2085	1833	102,483	<0.001	***

**Table 7:** ANOVA results for the optimal model for diabetes. The nonsignificant effects of private outdoor space and household overcrowding cannot be eliminated based on the AIC and, thus, remained in the final model after model selection. Variables with *p*-values < 0.001 are indicated with ***, variables with *p*-values < 0.01 with **, and variables with *p*-values < 0.05 with *. Final model AIC = 13,203, dAIC = 2.01.

	Degrees of Freedom (df)	Deviance	Residual df	Residual Deviance	Pr(>Chi)	Sig.
NULL			1841	226,135		
Dist_parks	1	5720	1840	220,415	<0.001	***
Private_outdoor_space	1	41	1839	220,375	0.463	
Dist_GP_services	1	959	1838	219,415	<0.001	***
Acceptable_GP_appointments	1	71,258	1837	148,157	<0.001	***
Dist_sports_or_leisure_facilities	1	308	1836	147,849	0.043	*
Household_overcrowding	1	33	1835	147,816	0.508	
Personal_crime	1	698	1834	147,118	0.002	**
Child_poverty	1	7183	1833	139,935	<0.001	***
Rough_sleeping	1	1691	1832	138,243	<0.001	***

**Table 8:** ANOVA results for the optimal model for dementia. The nonsignificant effects of personal crime cannot be eliminated based on the AIC and, thus, remained in the final model after model selection. Variables with *p*-values < 0.001 are indicated with ***, variables with *p*-values < 0.01 with **, and variables with *p*-values < 0.05 with *. Final model AIC = 224,933, dAIC = 0.918.

	Degrees of Freedom (df)	Deviance	Residual df	Residual Deviance	Pr(>Chi)	Sig.
NULL			1841	238,579		
Dist_parks	1	510	1840	238,069	0.042	*
Acceptable_GP_appointments	1	8635.5	1839	229,434	<0.001	***
Dist_sports_or_leisure_facilities	1	2118	1838	227,316	<0.001	***
Air_pollution	1	1055.5	1837	226,260	0.003	**
Personal_crime	1	154.8	1836	226,105	0.261	
Child_poverty	1	844.4	1835	225,261	0.009	**

**Table 9:** ANOVA results for the optimal model for kidney and liver disease. Variables with *p*-values < 0.001 are indicated with ***, variables with *p*-values < 0.01 with **, and variables with *p*-values < 0.05 with *. Final model AIC = 13,295, dAIC = 0.965.

	Degrees of Freedom (df)	Deviance	Residual df	Residual Deviance	Pr(>Chi)	Sig.
NULL			1841	181,197		
Dist_parks	1	5603.3	1840	175,594	<0.001	***
Private_outdoor_space	1	1263.9	1839	174,330	<0.001	***
Acceptable_GP_appointments	1	25,666	1838	148,664	<0.001	***
Air_pollution	1	475.5	1837	148,188	0.014	*
Child_poverty	1	1875.5	1836	146,313	<0.001	***
Rough_sleeping	1	789.2	1835	145,524	0.002	**

**Table 10:** ANOVA results for the optimal model for musculoskeletal conditions. Variables with *p*-values < 0.001 are indicated with ***, and variables with *p*-values < 0.01 with **. Final model AIC = 12,719, dAIC = 0.54.

	Degrees of Freedom (df)	Deviance	Residual df	Residual Deviance	Pr(>Chi)	Sig.
NULL			1841	187,722		
Dist_parks	1	2486	1840	185,236	<0.001	***
Private_outdoor_space	1	18,777	1839	166,459	<0.001	***
Dist_GP_services	1	4203	1838	162,256	<0.001	***
Acceptable_GP_appointments	1	48,142	1837	114,113	<0.001	***
Air_pollution	1	3297	1836	110,816	<0.001	***
Household_overcrowding	1	1995	1835	108,821	<0.001	***
Child_poverty	1	463	1834	108,359	0.005	**
Rough_sleeping	1	2051	1833	106,307	<0.001	***

**Table 11:** ANOVA results for the optimal model for respiratory conditions. The marginally significant effects of distance to parks cannot be eliminated based on the AIC and, thus, remained in the final model after model selection. Variables with *p*-values < 0.001 are indicated with ***, and variables with *p*-values < 0.01 with **. Final model AIC = 12,921, dAIC = 1.65.

	Degrees of Freedom (df)	Deviance	Residual df	Residual Deviance	Pr(>Chi)	Sig.
NULL			1841	178,215		
Dist_parks	1	219	1840	177,995	0.066	
Dist_GP_services	1	6442	1839	171,553	<0.001	***
Acceptable_GP_appointments	1	41,035	1838	130,518	<0.001	***
Air_pollution	1	7741	1837	122,777	<0.001	***
Household_overcrowding	1	469	1836	122,308	0.007	**
Personal_crime	1	613	1835	121,695	0.002	**
Rough_sleeping	1	3008	1834	118,687	<0.001	***

## Data Availability

The data set compiled here is available upon request from the corresponding author.
